# Reduction of radiation pneumonitis by V20-constraints in breast cancer

**DOI:** 10.1186/1748-717X-5-99

**Published:** 2010-10-29

**Authors:** Ulla Blom Goldman, Berit Wennberg, Gunilla Svane, Håkan Bylund, Pehr Lind

**Affiliations:** 1 Department of Oncology, Karolinska University Hospital, Stockholm, Sweden; 2 Department of Hospital Physics, Karolinska University Hospital, Stockholm, Sweden; 3 Department of Radiology, Karolinska University Hospital, Stockholm, Sweden; 4 Department of Radiology, Ersta Hospital, Stockholm, Sweden; 5 Karolinska Institutet Stockholm, Sweden

## Abstract

**Introduction:**

Adjuvant local-regional radiotherapy (LRRT) is routinely recommended for breast cancer patients. It is well known being related to pulmonary side-effects. We studied post-RT radiological changes on X-ray and CT, and correlated the findings with Quality of Life (QoL), common dosimetric factors and co-variates. The results were compared with a previously reported cohort of 137 irradiated women.

**Methods:**

88 women underwent chest X-ray and CT pre-and 4-5 months after 3-D planned LRRT, minimizing the dose to the ipsilateral lung to V_20 _< 30%. The lung field was divided into 3 regions and the development of post-RT density changes were graded (0-3). Patients with radiological changes were compared with non-responders. Clinical symptoms were registered and data on patient and treatment related co-variates were gathered prospectively. The ipsilateral lung dosimetric factors V_13_, V_20_, V_30 _and mean dose were calculated and QoL was assessed before and 4 months after RT.

**Results:**

The use of dose-volume constraints significally reduced moderate-severe radiological changes on chest X-ray compared with our earlier study (Chi square trend test: p < 0.001). Symptomatic pneumonitis was also rare in the present study. No agreement was found between CT and chest X-ray as diagnostic tools for post-RT pneumonitis. V_13 _correlated independently with radiological changes on CT (logistic regression: p = 0.04; ROC area: 0.7). The Co-variates smoking habits, age, chemotherapy, endocrine or trastuzumab therapy did not influence the outcome on multivariate analysis. QoL changes in physical function, i.e. fatigue, dyspnoea were not detected but there was a trend for a worse recovery after chemotherapy in patients with high V_13 _(Spearman Rank Correlation: p < 0.05).

**Conclusions:**

The use of dose-volume constraints significantly reduced post-RT radiological changes on chest X-ray in LRRT for BC. The lung changes on CT were also generally limited when we used this strategy and was not always picked up on chest X-ray. Variation in V_13 _alone was correlated with occurrence of lung changes on CT.

## Introduction

Postoperative radiotherapy (RT) for breast cancer (BC) plays an important role for reducing the rates of local recurrence and death [1-3]. The treatment, however, delivers some unwanted irradiation to the lung and heart. Side-effects to the lungs are in the form of acute pneumonitis and sub acute/late lung fibrosis. The risk for acute and chronic RT-induced lung morbidity is influenced by total dose, dose per fraction and irradiated lung volume. When a 3-D RT-planning technique is used, it is possible to quantify and limit the amount of individually irradiated lung volume. Clinical data suggest that a total lung dose of more than 20 Gy given with conventional fractionation should be avoided if the unirradiated lung volume is not sufficient to guarantee essential breathing function [4]. In our previous work, we found no case of moderate symptomatic radiation pneumonitis (RP) in patients who received doses ≥ 20 Gy (V_20_) to less than 30% of the ipsilateral lung volume [5]. We therefore used this cut-off level in the present trial. Other groups have found relations between chemotherapy [6,7] and tamoxifen intake [8] and RT-induced lung toxicity. In previous studies we have also found an association with age [5,9]. Individual sensitivity to irradiation is also known but a rare genetic condition in the population[10]. However it is shown that possessions of specific genes variants is predictive for the development of adverse effects after radiotherapy [11-13]. In contrast smoking has been reported to reduce the risk of RT-induced pneumonitis [14]. Side-effects to the normal lung tissue can occur as early as 6 weeks from the start of RT with symptoms of fever, dyspnoea and cough [15]. Signs of interstitial pulmonary inflammation can be detected on chest radiography (X-ray) in the irradiated lung. A later phase with fibrosis can be detected from 20 weeks and after about 36 weeks stationary fibrosis is obtained [16,17].

This study was performed to evaluate radiological pneumonitis (RP) on X-ray and CT in irradiated breast cancer women when the lung dose-volume constraints of V_20 _< 30% was used and to correlate the findings with common dosimetric factors (ipsilateral V_13_, V_20_, V_30_, MLD), Quality of life (QoL)-effects, symptoms and co-variates and compare the outcome to a previously reported study of 137 irradiated women [9].

## Methods

This study was approved by the local ethics committee. Participating women gave informed consent before study enrolment.

### Study population

All women who were referred to the Radiotherapy Department at Stockholm Söder Hospital during 2003-2005 for adjuvant LRRT after surgery for early breast cancer were asked to participate in this trial. Ninety-five patients were included, but seven patients withdrew their consent due to early relapse and were not evaluable. Eighty-eight patients were thus followed for seven months after RT for symptoms of acute/subacute radiation induced pulmonary complication. Mastectomy was done in 69 patients, while 19 patients were operated with conservative breast surgery. Seventy-two patients were irradiated with LRRT to the chest wall or breast, axilla and supraclavicular region and in these patients the internal mammary lymph nodes (IMN) were included. A total of 16 patients received RT excluding the IMN, i.e. 9 patients were given irradiation to the breast, axilla and supraclavicular region and 7 patients were referred for RT to the axilla and supraclavicular fossa only.

The mean age of the patients was 56 years (range 32-81). Data on potential confounding factors were collected prospectively, i.e. history of cardio vascular or pulmonary co-morbidity, smoking habits, functional level (i.e. not being able to climb three flights of stairs without a rest due to shortness of breath) and adjuvant hormonal -, trastuzumab- and chemotherapy treatment.

The chemotherapy was concluded 3-4 weeks prior to RT. Concurrent chemotherapy was never given. The typically regime consisted of doxorubicin, cyclophosphamide and 5-fluorouracil, but in 28 patients the therapy included docetaxel. Five patients received trastuzumab during RT. Intake of tamoxifen and anastrozol during RT was evenly split among the women.

To asses and evaluate Quality of life (QoL) before and after RT we used the EORTC QLQ-C30 version 3.0 and the EORTC QLQ-BR23 questionnaires.

### Radiotherapy treatment techniques

The used RT treatment techniques are described in detail in an earlier publication [18]. LRRT after mastectomy was delivered with an anterior electron beam covering the chest wall and the IMN (range 6-12 MeV) and with a 6 MV photon beam covering the supraclavicular region. LRRT after partial mastectomy consisted of two tangential photon beams of 4 or 6 MV including the breast parenchyma (50 Gy) and the regional lymph nodes were treated in a similar way as described above (46 Gy). An additional oblique electron beam was added to include the IMN (46 Gy) in four cases. The prescribed dose was given in daily fractions of 2 Gy, five days a week. In the present study all patients underwent 3-D dose treatment planning (Pinnacle; version 6.2b) with avoidance of a dose exciding 20 Gy to more than 30% of the ipsilateral lung volume but with a good coverage of the clinical target volume (CTV). The cumulative dose-volume histograms were calculated and the ipsilateral lung volume receiving ≥ 13 Gy (V_13_), >20 Gy (V_20_), >30 Gy (V_30_) and mean lung dose were defined.

### Monitoring for symptomatic pneumonitis and evaluation of radiological pneumonitis on X-ray and CT with the Arrigada's classification

All patients were followed for respiratory symptoms, i.e. cough, dyspnoea with or without fever, 1, 4 and 7 months after the termination of RT. The patients were classified into three groups according to CTC-criteria (version 2.0) [19].

0. No complications: no registered respiratory symptoms monitored by the clinician.

1. Mild reaction: cough and/or dyspnoea with our without fever judged to be radiation induced.

2. Moderate reaction: same as 1 but with impaired daily functions and treated with corticosteroids.

CT of the thorax was performed before and 4 months after RT and standardized chest X-ray was conducted after 5 months and evaluated by the same specialist in diagnostic radiology (HB) as in our previous trial [5]. The reproducibility of this scoring system was validated in our earlier publication [5].

On the frontal chest radiograph the lung was divided into the three regions suggested by Arrigada, i.e. the apical-lateral (A-L), central-parahilar (C-P) and basal-lateral (B-L) regions [19]. The border between the A-L and B-L regions was set at the level of the pulmonary artery. The width of the C-P region was set to 5 cm and the upper and lower border were set two vertebrae above and below the level of pulmonary artery, respectively. Radiological pneumonitis (RP) was quantified according to Arrigada's classification. The highest-density grade in each region, i.e. 0 = no evidence of fibrosis, 1 = linear streaks, 2 = moderate opacification, 3 = complete opacification were added together to form total scores ranging from 0 to 9. Total scores of 1-3 were considered to represent slight radiological RP and score of 4-9 moderate to severe RP. This method has been described in detail in our earlier study [5].

### Evaluation of Quality of life

We used the European Organisation for Research and Treatment of Cancer (EORTC) form QLQ-30 (version 3.0) [20] and the EORTC QLQ-BR-23 to asses QoL [21].The forms were completed at baseline prior to and 4 months after RT. The QLQ-30 questionnaires consist of a total of 30 items. Five functional scales (physical, role, cognitive, emotional and social); nine symptom scales (fatigue, nausea/vomiting, pain, dyspnea, insomnia, appetite loss, constipation, diarrhea and financial difficulties) and one Global health status. QLQ-BR23 includes 23 items assessing disease symptoms, therapy side effects such as breast, arm symptom, hair loss, body image, sexual functioning, sexual enjoyment and future perspective.

Eighty-one patients completed both measurements. In seven cases the pts did not receive the 2^nd ^form. The form was double-sided and in some cases not completed on the back page. Scoring was performed in according with the EORTC scoring manual. Statistics and missing data were handled according to the manual. A four-point response scale was used to asses each item concerning functions or symptoms from 1 (not at all) to 4 (very much), and a seven-point scale was used for global health status/QoL from 1 (very poor) to 7 (excellent). The scale scores were linearly transformed into scores of 0-100 according to the EORTC manual. A high score on the global health status/functional scale represents a high/healthy level of functioning. In contrast a high score on the symptom scale represents a high level of symptomatology/problems. A study of the subjective significance of changes in QoL scores has suggested that a mean change of 5 to 10 on the multi-item scales is perceived as little change, 10 to 20 as moderate change and greater than 20 as very much change. Greater than ten points on the transformed questionnaire scale were considered clinically meaningful [22,23].

In the present paper, three functional scales (physical functioning, role functioning and social functioning) and four symptom scales (fatigue, pain, dyspnoea and insomnia) were included from QLQ C-30. In the EORTC QLQ-BR23 form we included functional scales (future perspective).

### Statistical methods

The relation between symptomatic and radiological RP and the relation between radiological RP and the dosimetric factors and co-variates was analyzed with univariate and multivariate logistic regression (Wald-Enter method). Chi square trend test was used for test of correlation between radiological RP on X-ray in the present and earlier studies. To test agreement between CT-and X-ray for the diagnosis of radiological RP, Kappa-statistics was used.

Receiver operating characteristics curves (ROC) were used to predict radiological RP with V_13 _[24]. Changes in QoL-scores in relation with V_13 _were evaluated with Spearman Rank Correlation. All reported results were based on two-sided tests and p-values < 0.05 were considered statistically significant.

## Results

### Radiological and symptomatic radiation pneumonitis

**Figure 1:**
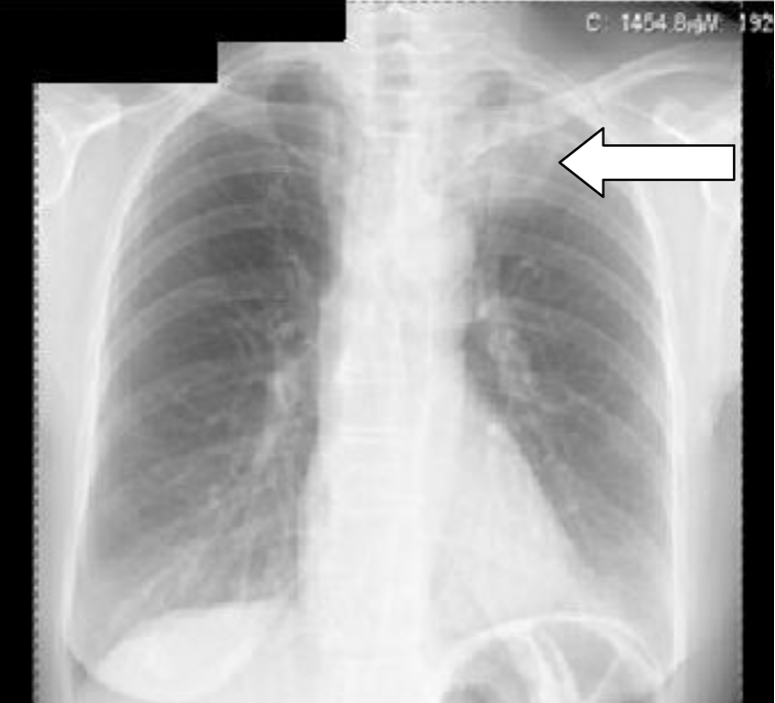
**Example of grade 3 RP in the apical-lateral region of the left lung on chest x-ray**. (= total score 3).

Figure 1 shows an example of post-RT radiological RP of grade 3 in the apical-lateral region of the left lung on chest X-ray (= total score 3). Symptomatic pneumonitis was very rare in this study. Only one patient developed a moderate reaction and was treated with corticosteroids and antibiotics, mild reactions were detected in 6 patients. There was, furthermore, no relation between symptomatic RP and radiological RP on chest X-ray or CT. Minor changes are not seen on chest X-ray, in due to that CT is a more sensitive method than X-ray to detect small effected areas of pulmonary changes.

**Figure 2:**
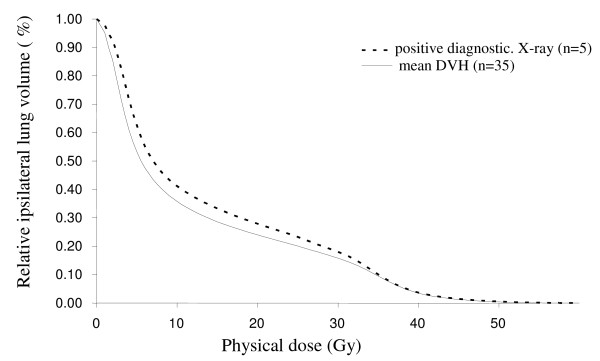
**Mean lung dos volume histograms (DVH) in patients with or without RP on chest X-ray**.

The use of dose-volume constraints significally reduced moderate-severe radiological RP on X-ray compared with the earlier treatment series for the technique with LRRT + IMN (Chi square trend test p < 0.001) (Table 1). There was, however, no difference when we compared the outcome for the technique LRRT-IMN in the present series with the previous trial (Table 1). The mean V_20 _for responders and non-responding patients are shown in Figure 2. The average V_20 _and MLD in our previous study was 35% and 16 Gy, respectively [25]. We found no correlation between any dosimetric factor or the studied co-variates and RP on chest X-ray (score 0 vs score 1-9) (logistic regression). In the preceding univariate analysis there was a borderline relation with radiological RP and anastrazol but this relation was thus not detected on the subsequent multivariate analysis which included the dosimetric factors and other co-variates.

**Table 1:** Relation between radiological changes and treatment techniques in the present and previous studies

Technique	LRRT+ IMN; n			LRRT-IMN; n		
Arrigada's classification scores	0	1-3	4-9	0	1-3	4-9

Present study	60	11	1	5	4	0

Previous study	58	38	20	12	9	0

	Chi square trend test p < 0.001			Chi square trend test p = 0.9		

There was no agreement between X-ray and CT as diagnostic tools for post-radiological RP, (Kappa test) (Table 2). V_13 _was most strongly and independently related with radiological changes on CT (score 0 vs 1-9) (logistic regression: p = 0.04; ROC-area: 0.7) [24]. No other factor was related to RP on CT. Table 3 shows the correlation between the dosimetric factors in this study. V_13 _was stronger correlated to MLD than V_20._

**Table 2:** Relation between radiological scores on X-ray and CT in the present series

	CT score			
		
X-ray score	0	1-3	4-9	Total
0	13	25	12	50

1-3	1	7	3	11

4-9	0	1	0	1

Total	14	33	15	62

Kappa statistics: p = 0.3

**Table 3:** Correlation between lung dosimetric factors in breast cancer irradiation

		V13	V20	V30	Mean
V13	Pearsson Correlation	1	.925**	.619**	.975**
	Sig. (2-tailed)		.000	.000	.000

V20	Pearsson Correlation	.925**	1	.820**	.926**
	Sig. (2-tailed)	.000		.000	.000

V30	Pearsson Correlation	.619**	.820**	1	.687**
	Sig. (2-tailed)	.000	.000		.000

Mean	Pearsson Correlation	.975**	.926**	.687**	1
	Sig. (2-tailed)	.000	.000	.000	

** Correlation is significant at the 0.01 level (2-tailed)

### Quality of life

Most of the side effects from RT appeared to have little effect on QoL in the present trial. Chemotherapy was concluded 3-4 weeks prior to RT and the patients started with a higher score on fatigue at baseline due to this.

The variables role functioning, social functioning and future perspective, were improved 4 months after RT compared to baseline (Table 4). Physical functioning, appeared not to be affected by RT. There were no changes for pain and dyspnoea after RT in this series.

**Table 4:** Pre and post RT Quality of Life EORTC scores in breast cancer irradiation

	Mean QLQ-30 scale values before and after RT and for paired difference					
	n	Before RT	After RT	Difference	95% CI	P-value
**Functional scales**						
Physical functioning	81	78.5	80.6	2.1	-1.2-5.4	0.20
Role functioning	80	62.3	70.6	8.3	1.3-15.4	0.021
Social functioning	73	70.6	76.3	5.7	0.7-10.7	0.026

**Symptom scales**						
Fatique	73	37.1	31.7	-5.5	-10.0 - -1.0	0.018
Pain	81	24.7	24.3	-0.4	-5.3 - 4.4	0.87
Dyspnoea	79	29.1	28.3	-0.8	-6.9 - 5.3	0.78
Insomnia	81	36.6	41.6	4.9	-1.5 - 11.3	0.13

	**Mean QLQ-BR23 scale values before and after RT and for paired difference**					
	**n**	**Before RT**	**After RT**	**Difference**	**95% CI**	**P-value**

**Functional scales**						
Future perspective	77	44.2	53.7	9.5	3.4-15.6	0.003

Patients with high V_13 _appeared however not recover equally well. However, insomnia showed a trend to increase after RT (Table 4). When changes in the individual QLQ-variates fatique and dyspnoea were related to V_13 _(Spearman correlation) there was thus a negative correlation. There was a significant correlation between high V_13 _and difficulties to take short walks, which could be of clinical significance, and the correlation was reported also when patients rated there overall total quality of life during the last week.

## Discussion

Clinically significant radiological and symptomatic RP was rare in this study when 3-D treatment planning, aiming at minimizing V_20 _to the ipsilateral lung to <30% was used for LRRT in early breast cancer. The result indicates that the used dose-volume constraints significantly reduced moderate-severe radiological RP on chest X-ray, in the present series, compared to our previous study [25]. The lung changes could not always be detected on chest X-ray and were also infrequent and generally limited on CT when this strategy was used. Variation in dosimetry alone (V_13_) was correlated with occurrence of radiological RP on CT. ROC analyses was performed, yet the area under the curve was only 0.7 which is not an ideal predictive value [24]. Co-variates as smoking habits, age, exposure to chemotherapy, endocrine- or trastuzumab therapy did not influence the outcome, but the few events may have hampered the possibility to evaluate this. In the present study, some women received radiation to the internal mammary nodes (IMN). Whether the IMN need to be included in the CTV is not fully known. In the last years, many RT centers have excluded RT to the lower IMN, in order to avoid cardiac and lung toxicity. The meta-analysis in Lancet, 2005, however demonstrated a benefit for post mastectomy RT in women with positive LN and the majority of these women received RT to the lower IMN (21 of 23 studies) [1].

The LRRT-IMN group of our present series included only nine patients. We used the same RT-technique in both this and the previous study, and as could be expected, there was no difference in radiological RP (Table 1). CTV volumes minus IMN usually give lower doses to the lung. Limiting the IMN irradiation to the three upper intercostals spaces also lower the dose to the heart. It is probably of great importance to reduce radiation to organs at risk like heart and lung, when adjuvant treatment is given. The average patient has a long expected survival, but as there are many new systemic therapies which may interact with RT this can lead to additional side-effects. Aromatase Inhibitors (AI) have replaced tamoxifen in many postmenopausal patients.

The AI treatment in combination with RT is investigated in a randomised trial presented in Lancet Oncol. 2010. The results suggested that AI can be used early, but there still are doubts on potential long-term toxic effects, mainly cardiac in combination with RT [26,27].

Genetic factors may also play a vitale role in treatment. By identifying genetic factors associated with radiosensitivity it will be easier to predict which patients are at increased risk for complications secondary to radiation treatment [11-13].

Even though symptomatic and radiological RP were rare in our trial, they still could be of importance if they prevail, as late changes could increase the risk of secondary lung cancer. This increased risk is seen in smokers five years after RT [28].

To improve radiotherapy techniques and continue to study pulmonary morbidity and QoL after RT, is of great importance as breast cancer is a common disease among women.

In conclusion, V20-constraints significantly reduced post-RT radiological changes on chest X-ray in LRRT for breast cancer. Symptomatic pneumonitis was, furthermore, rare in the present study when this strategy was used. There was no agreement between X-ray and CT as diagnostic tools for post-RT in this trial, as the lung changes typically were too limited for detection on X-ray. V_13 _was most strongly related to radiological RP on CT. V_13 _was stronger correlated to MLD than V_20_, and may be an important metric in future trials on RT-induced lung toxicity.

## Competing interests

The authors declare that they have no competing interests.

## Authors' contributions

UBG coordinated the study, collected the data and drafted the manuscript.

UBG, BW, GS and PL were involved with the design of the study.

HB and GS analysed X-ray and CT diagnostics. BW analysed RT-doses. PL supported with the statistics. All authors read and approved the final manuscript.
